# Dietary acrylamide exposure was associated with mild cognition decline among non-smoking Chinese elderly men

**DOI:** 10.1038/s41598-017-06813-9

**Published:** 2017-07-25

**Authors:** Zhao-min Liu, Lap Ah Tse, Bailing Chen, Suyang Wu, Dicken Chan, Timothy Kowk, Jean Woo, Yu-Tao Xiang, Samuel Yeung-shan Wong

**Affiliations:** 1 Department of Nutrition, School of Public Health, Sun Yat-sen University, Guangzhou, China; 2 Division of Environmental and Occupational Health, Jockey Club School of Public Health and Primary Care, the Chinese University of Hong Kong, Hong Kong SAR, China; 3 Department of Spine Surgery, The First Affiliated Hospital of Sun Yat-sen University, Guangzhou, PR China; 4 Division of Family Medicine and Primary Care, Jockey Club School of Public Health and Primary Care, the Chinese University of Hong Kong, Hong Kong SAR, China; 5 Department of Medicine and Therapeutics, the Chinese University of Hong Kong, Hong Kong SAR, China; 6 Unit of Psychiatry, Faculty of Health Sciences, University of Macau, Macao SAR, China

## Abstract

The aim of the study is to explore the longitudinal association of dietary acrylamide exposure with cognitive performance in Chinese elderly. The analysis was conducted among 2534 non-smoking elderly men and women based on a prospective study, Mr. and Ms. OS Hong Kong. Dietary acrylamide intake was assessed by food frequency questionnaires with data on local food contamination, derived from the first Hong Kong Total Diet Study. Global cognitive function was assessed by Cantonese version of Mini-Mental State Exam (MMSE) at the baseline and the 4^th^ year of follow-up. Multivariable-adjusted linear and logistic regression models were used to assess the associations of dietary acrylamide with MMSE score changes or risk of poor cognition. The results indicated that among men with MMSE ≥ 18, each one SD increase of acrylamide decreased MMSE score by 7.698% (95%CI: −14.943%, −0.452%; p = 0.037). Logistic regression revealed an increased risk of poor cognition (MMSE ≤ 26) in men with HR of 3.356 (1.064~10.591, p = 0.039). The association became non-significance after further adjustment for telomere length. No significant association was observed in women. Dietary acrylamide exposure was associated with a mild cognitive decline or increased risk of poor cognition over a 4-year period in non-smoking Chinese elderly men.

## Introduction

Ageing is associated with loss of cognition and an increased risk of dementia. Impaired cognition is the leading cause of loss of independence in daily activities^1^, hospitalization^2^, and mortality among elderly^3^, which have placed growing demands on health and long-term care providers. Identification of modifiable factors that could prevent cognitive decline is essential to improve the autonomy and quality of life of older people.

The major sources of acrylamide in human were originally regarded as occupational exposure and smoking^4^. However, the findings of significant amounts of acrylamide produced from commonly consumed carbondrate-rich foods during cooking raised great health concerns. Acrylamide in diet is formed when reducing sugars (glucose or fructose) and amino acids (asparagine) react with each other during high temperature cooking such as toasting, frying or baking. Fried potato products, bread, biscuits, roasted cereals, and coffee showed the highest levels of acrylamide in diet^5^. Acrylamide was classified as a probable human carcinogen by the International Agency for Research on Cancer (IARC) on the basis of its carcinogenicity in rodents^6^. In addition to its carcinogenicity and genotoxicity, acrylamide also possess the hazardous property of neurotoxicity^7^. Acrylamide and its bio-transformed metabolite, glycidamide, are neurotoxins in both animals and humans^8^.The neurotoxicity involves not only the peripheral but also the central nervous system^9^. As a neurotoxin, elderly exposure to acrylamide is of particular concern on cognitive performance. Animal experiments have shown, repeated exposure to acrylamide has been shown to induce degeneration of nerve terminals in brain areas critical for learning, memory and other cognitive functions (i.e., cerebral cortex, thalamus, and hippocampus)^10, 11^.

The neurotoxic effects of acrylamide were often observed in humans at high levels of exposure in occupational settings such as gel chromatography or waste water management^12, 13^. There are controversies whether relatively low levels of acrylamide exposure (0.8–3 μg/kg/day, FAO/WHO report 2002) in the diet could result in clinical neuropathy. Animal studies have reported a similar neurotoxic effect observed at low and high doses of acrylamide with the low doses simply requiring longer exposure^14^. A recent animal study^10^ in rats with daily exposure to acrylamide from prenatal throughout the lifespan indicated acrylamide, when provided chronically at relatively low doses for 8 months, reduced learning task performance. However, the neurotoxicity and mechanisms of acrylamide from diet on cognitive function have not been elucidated in epidemiological studies, especially among elderly who are free of occupational exposure but at an increased risk of cognitive decline due to aging.

Most of previous observational studies of acrylamide focused on assessment of peripheral nerve dysfunction such as numbness in the hands and feet, fatigue of the lower limbs, unsteady walking, but studies on the disorders in central brain function and cognition were rare. In addition, long-term dietary acrylamide exposure has been related to aging-associated cognitive impairment, possibly due to enhanced inflammation. Leukocytes with longer telomere length (TL) are more responsive to inflammatory stimuli^15^, yet TL has not been evaluated in relation to acrylamide and cognition. Our aim was to provide longitudinal evidence on the association between dietary acrylamide intakes, assessed using food contaminant data from a local Total Diet Study, and cognitive performance among Chinese elderly.

## Methods

### Participants

Four thousand community-dwelling men and women aged 65 years or over were invited to attend a health check carried out in the School of Public Health of the Chinese University of Hong Kong between August 2001 and December 2003 by placing recruitment notices in community centers and housing estates. Only ethnical Chinese subjects were recruited. Those who were unable to walk without assistance, had bilateral hip replacement or were physically or mentally incapacitated to give informed consent or participate were excluded. The recruitment has been described in more details elsewhere^16^. The study was approved by the Clinical Research Ethics Committee of the Chinese University of Hong Kong and complied with the Declaration of Helsinki. The informed consent for this study was obtained before study enrollment and during the data collection.

Eligible participants were invited to the research center for baseline assessment. They were interviewed using a structured and standardized questionnaire and underwent physical examination. The questionnaires included demographic information, socioeconomic status, family and medical history, current medications, smoking and drinking of alcohol, tea and coffee, dietary factors and physical activities. Follow-up examination was conducted four-year later.

### Dietary intake and acrylamide exposure assessment

Dietary intakes were assessed by a validated food frequency questionnaire (FFQ)^17^. The FFQ containing 329 food items was used to estimate dietary intakes at baseline. Consumption frequencies were recorded with a 7-item scale from “never” to “more than once a day”. The frequency was generated from the midpoint of the categories. Portion sizes were estimated using natural units, household measures, or grams with the aid of a set of photographs. To calculate the consumption of each food item, the portion consumed was multiplied by the frequency. Total energy and nutrients intakes were calculated based on the Chinese Food Composition Table of 2004.

Individual acrylamide exposures (in µg/day) in diet were calculated by multiplying the frequency of consumption of each food item by its mean acrylamide content per serving (in µg/g). Food contamination data on acrylamide were derived from the first Hong Kong Total Diet Study (TDS)^18^ by the Hong Kong Health Department. Details on sampling methodology of the TDS have been published elsewhere^19^. To combine individual consumption data with contamination data, all the food items in the FFQ were linked with the closest food items of the TDS. In the Hong Kong TDS, a total 150 TDS food items were determined and prepared into table-ready forms on four occasions, and they were then combined into 600 composite samples for laboratory analysis.The foods that were used in the acrylamide intake assessment were assigned the mean value of the acrylamide levels detected in the TDS food items or a value of one-half the quantitation limit when concentrations were lower than the quantitation limit^18^.

### Cognition assessment by Mini Mental State Examination (MMSE)

Cantonese version of Mini-Mental State Examination (MMSE)^20^ was used to evaluate global cognitive functions. MMSE was assessed at baseline and re-assessed at the fourth year in several cognitive domains, including orientation, immediate and short-term recall, attention and calculation, word finding, construction reading and writing skills, and ability to follow a three step command. The MMSE is a validated method of assessing global cognitive function that is widely used in clinical practice and research and is an effective screening tool for cognitive impairment in older adults^21^. It can be used by clinicians to help diagnose dementia and assess its progression and severity. Scores for the MMSE range from 0 to 30, with higher scores indicating better cognitive function.

### Telomere length (TL)

Telomere length (TL) measurement followed the method published by Cawthon^22^ with modifications by obtaining a corrected Ct ratio of telomere and control gene (36B4) from quantitative real-time PCR (T/S ratio). In brief, DNA was extracted in the peripheral blood by the phenol–chloroform method using a QiAmp DNA blood kit (Qiagen, Germantown, MD, USA) and stored at −80 °C. Quantitative real-time PCR was performed by Roche Light Cycler 480 (Roche, Mannheim, Germany).

### Other covariates

Physical activity was measured by the Physical Activity Scale for the Elderly Questionnaire (PASE)^23^. Height was measured by the Holtain Harpenden standiometer (Holtain Ltd., Crosswell, UK). Body weight was measured with the subjects wearing a light gown by the Physician Beam Balance Scale (Healthometer, IL, USA).

### Statistical analysis

All analyses were carried out with SPSS statistical software version 21.0 (SPSS, Inc., Chicago, Illinois). A 2-sided p value of less than 0.05 was considered significant. In order to restrict the influence of smoking on acrylamide exposure, our analysis was conducted among 2534 non-smoking participants. Thus, diet is the main source of exposure to acrylamide in non-smokers^24^. Independent t-test and Chi-square tests were used for continuous and categorical variables respectively to compare the baseline characteristics between men and women. The associations of dietary acrylamide exposure and cognition were tested using both linear regression and logistic regression models by controlling for relevant confounders. Linear regression was used to test the association of dietary acrylamide exposure and the 4-year score changes and percentage changes of MMSE. Daily acrylamide exposure was log_10_-transformed because of a right-skewed distribution. The analyses were conducted separately for both sexes and the whole participants. To reduce reporting bias due to impaired cognitive functioning, we re-analyzed the data after excluding participants with MMSE score <18 at baseline. Logistic regression models were used to calculate hazards ratios (HRs) and 95% confidence intervals (CIs) between acrylamide intakes (continuous value) and risk of impaired cognition decline (MMSE ≤ 24). We repeated the analysis with adjustment of MMSE cutoff to 26 (MMSE ≤ 26) in consideration of age and education^25^. To evaluate whether TL mediated the association of acrylamide exposure with cognitive function, we added TL in the regression model. In sensitivity analyses, we excluded patients of diabetes, stroke and heart diseases because the chronic diseases may induce changes in diet and lifestyle. Effect modification by obesity (body mass index (BMI) < vs. ≥ 24 kg/m^2^) and education (below vs above university) was tested by including an interaction term of these variables with the acrylamide exposure in fully adjusted regression models. A worst-case analysis was also conducted with exclusion of participants with acrylamide exposure above 95% CI.

## Results

We excluded current and former smokers from all 4000 older adults recruited at baseline to rule out the influence of smoking, and retained 2534 nonsmokers (724 men and 1810 women) for the data analysis. The baseline characteristics of the participants were compared between men and women (Table 1). Compared with women, men had higher education, be more likely to be married or cohabitation, had higher prevalence of stroke, had higher dietary acrylamide exposure, higher total energy, coffee, alcohol and tea drinking, higher consumption of dietary fiber, carbohydrate, fruits and isoflavones than women. Men had higher MMSE scores at baseline and more declined cognition and shorter TL than women.

**Table 1:** Participants’ characteristics among non-smoking Chinese elderly men and women.

Participants’ characteristics	Men	Women	P
n	724	1810	
Age	71.7 ± 4.9	72.3 ± 5.3	0.01
Education above university (%)	20.2	6.5	0.001
Married or cohabitation (%)	90.3	54.8	0.001
Acrylamide intake (µg/d)	16.1 ± 7.5	13.3 ± 7.8	0.01
Medical history (%)			
Diabetes	15.5	14.3	0.557
Hypertension	41.9	44.4	0.239
Stroke	5.5	3.3	0.008
Heart diseases	9.4	9.4	0.999
Cancers	3.3	4.6	0.136
Dietary factors			
Energy intake (kcal/d)	2125 ± 606	1587 ± 464	0.001
Carbohydrate (g/d)	292.8 ± 89.7	227.1 ± 69.6	0.001
Coffee (ml/d)	21.4 ± 65.6	15.4 ± 54.9	0.001
Tea (ml/d)	555 ± 540	354 ± 429	0.001
Alcohol intake (g/day)	14.7 ± 64.4	1.61 ± 15.4	0.001
Fibre (g/d)	10.3 ± 5.2	8.9 ± 4.8	0.001
Fish and seafood (% of total energy)	4.9 ± 3.4	5.1 ± 4.1	0.50
French fries/potato chips(% of total energy)	0.30 ± 0.99	0.16 ± 0.95	0.001
Fruits (% of total energy)	8.0 ± 5.2	9.0 ± 5.5	0.06
Red and processed meats (% of total energy)	7.9 ± 5.4	6.3 ± 4.6	0.05
Total isoflavones (mg)	17.1 ± 24.6	12.7 ± 13.5	0.001
Whole grain (g/day)	54.5 ± 87.6	67.2 ± 83.3	0.43
Calcium supplements usage (%)	10.5	18.3	0.001
Total AHA scores	46.0 ± 9.6	47.0 ± 9.4	0.43
Body weight (kg)	62.4 ± 9.0	54.8 ± 8.4	0.01
Body mass index (BMI, kg/m^2^)	23.4 ± 3.0	24.0 ± 3.4	0.001
Systolic Blood Pressure (SBP, mmHg)	142.0 ± 20.1	143.6 ± 18.4	0.01
Diastolic Blood Pressure (DBP, mmHg)	78.6 ± 9.2	77.3 ± 9.2	0.03
PASE total score	99.3 ± 51.5	85.9 ± 33.3	0.001
MMSE at baseline	27.0 ± 2.9	24.3 ± 3.9	0.001
MMSE change at 4^th^ year follow-up	−0.47 ± 3.349	0.85 ± 3.446	0.001
DNA Telomere length	8.79 ± 1.63	14.37 ± 1.87	0.001

Data were presented as mean ± SD or number (%). Independent t-test and Chi-square test were used for continuous and categorical variables, respectively. PASE: Physical Activity Scale for the Elderly; Total AHA scores were estimated based on the adherence index of American Heart Association on dietary and life style recommendations. MMSE: questionnaire for Mini-Mental State Exam.

The results of linear and logistic regression on the associations of acrylamide exposure and cognition are shown in Tables 2 and 3, respectively. Linear regression results (Table 2) showed that increased acrylamide exposure was modestly but significantly associated with decreased MMSE scores (both changes and percentage changes). Stratification analysis by gender indicated that the association was marginally significant for males but not for females. This association was attenuated to non-significance when TL was added to the model as continuous variable in men. Among elderly men with MMSE ≥ 18, each one standard deviation (SD) increase of acrylamide, the MMSE will decrease 7.698% (95%CI: −14.943%, −0.452%; p = 0.037). Sensitivity analysis showed similar results when we excluded participants with a history of diabetes, stroke or cardiovascular diseases (Supplemental Tables 1 and 2). When dietary acrylamide exposure was treated as continuous variable, the fully adjusted model by logistic regression in Table 3 revealed an increased risk of poor cognition for both cutoffs of MMSE ≤ 24 and MMSE ≤ 26. For the cutoff of MMSE ≤ 26, the adjusted HRs were of 3.356 (1.064~10.591, p = 0.039) and 1.091 (0.531~2.240, p = 0.831) for men and women, respectively, with each increase of 1 µg/d acrylamide intake. No statistically significant interactions were detected for BMI and education. The worst-case analyses by exclusion of participants of acrylamide exposure above 95% CI suggested similar findings on the association of dietary acrylamide and cognition (see Supplemental Tables 3 and 4).

**Table 2:** Multivariable linear regression analyses between dietary acrylamide intake and changes of MMSE at the 4^th^ year follow-up among Chinese elderly men and women.

	Model 1 (crude)	P	Model 2 (full adjustment)	P
**MMSE 4-year change**	β (95% CI)		β (95% CI)	
Men (n = 723)	−0.650 (−1.908, 0.609)	0.311	−1.519 (−3.176, 0.138)	0.072
Women (n = 1809)	−0.313 (−1.085, 0.458)	0.426	−0.292 (−1.320, 0.737)	0.578
Both men and women(n = 2533)	−0.897 (−1.548, −0.245)	0.007	−0.664 (−1.323, −0.005)	0.048
Participants with MMSE ≥ 18				
Men (n = 718)	−0.577 (−1.842, 0.686)	0.371	−1.443 (−3.098, 0.211)	0.087
Women (n = 1709)	−0.450 (−1.229, 0.328)	0.257	−0.631 (−1.665, 0.402)	0.231
Both men and women (n = 2427)	−0.942 (−1.598, −0.286)	0.005	−0.820 (−1.486, −0.154)	0.016
**MMSE 4-year % change**				
Men (n = 723)	−2.346 (−7.306, 2.614)	0.353	−3.385 (−8.368, 1.597)	0.183
Women (n = 1809)	−1.749 (−5.327, 1.830)	0.338	−1.667 (−5.249, 1.895)	0.357
Both men and women (n = 2532)	−4.084 (−6.986, −1.182)	0.016	−2.870 (−0.6730, 0.991)	0.145
Participants with MMSE ≥ 18				
Men (n = 580)	−3.792 (−9.403, 1.818)	0.185	−7.698 (−14.943, −0.452)	0.037
Women (n = 1387)	−1.862 (−7.540, 3.816)	0.207	−1.862 (−7.540, 3.816)	0.520

Dietary acrylamide intakes were log_10_ transformed. Multivariable linear regression models were adjusted for age (y), sex (not included for gender specific analysis), education, income, physical activity (PASE total scores), body mass index (kg/m^2^), medical history of hypertension (yes/no), diabetes (yes/no), and coronary heart disease (CHD) (yes/no), dietary intake of carbonhydrate (g %kcal), fish (g/week), fruit and vegetables (g/1000 kcal), fiber (g/d) and isoflavones (mg/d), alcohol drinking (g/day), tea drinking (ml/wk), total AHA scores. Total AHA scores were estimated based on the adherence index of American Heart Association on dietary and life style recommendations. MMSE: questionnaire for Mini-Mental State Exam.

**Table 3:** Hazard ratios (95% CI) of impaired cognition (MMSE ≤24 and ≤26) at 4^th^ year follow-up by dietary acrylamide exposure among non-smoking Chinese elderly men and women.

	Model 1 (crude)	P	Model 2 (full model)	P
	HR (95% CI)		HR (95% CI)	
**4** ^**th**^ **y MMSE** ≤ **24 as cutoff**				
Men (n = 590)	1.015 (0.984, 1.047)	0.337	1.029 (0.996, 1.062)	0.085
Women (n = 1381)	0.995 (0.979, 1.012)	0.593	1.006 (0.989, 1.024)	0.498
**4** ^**th**^ **y MMSE** ≤ **26 as cutoff**				
Men (n = 592)	1.226 (0.451, 3.330)	0.290	3.356 (1.064, 10.591)	0.039
Women (n = 1443)	0.596 (0.333, 1.069)	0.083	1.091 (0.531, 2.240)	0.831

Data analysis was conducted by logistic regression model. Hazard rations (HR): Risk of MMSE ≤24 or ≤26 with an increase of 1 µg/d acrylamide intake. Adjusted variables included age (y), education, PASE total scores, dietary carbohydrate intake (% total energy), total AHA scores, baseline body weight, coffee (ml/d), tea drinking (ml/d), alcohol drinking (ml/d), medical history of diabetes (yes/no), stroke (yes/no), hypertension (yes/no), heart infarction (yes/no), any cancers (yes/no), total isoflavoens intake (mg/d), fruit and vegetables intakes (g/1000 kcal), fish consumption (g/1000 kcal). Total AHA scores were estimated based on the adherence index of American Heart Association on dietary and life style recommendations. MMSE: questionnaire for Mini-Mental State Exam.

## Discussion

In this cohort of non-smoking Chinese elderly, dietary exposure to acrylamide, even at a relatively modest level as assessed based on local food contaminant data, was associated with a mild decline in cognition, or increased risk of poor cognition over a 4-year period in men but not in women. TL may be mediated the association. To our knowledge, this is the first longitudinal study exploring the association of dietary acrylamide exposure and cognitive performance in elderly population. Given the widespread presence of acrylamide in food and the relatively common nature of cognitive decline in elderly due to aging, even a modest association between the two would have important public health implications. Studies have shown even one point increase in MMSE score has been associated with a significantly decreased risk of onset of any activities of daily living limitation^26, 27^. Our observation suggested that acrylamide may precede subtle cognitive decline in the future.

The average acrylamide exposure in our older participants was 0.27 µg/d for men and 0.24 µg/d for women. The amounts are similar with the Chinese general population^28^, but were in the lower part of the WHO estimate range of 0.3–0.8 μg/kg bw/day for developed countries^19^. This could be due to Chinese elderly had far less fried and baked foods intakes than their western counterparts^29^. In our analysis, the association between acrylamide intake and cognition was more obvious for men than women. Men and women have specific biological characteristics that may partly explain differences in cognition^30^. However, there is no biological hypothesis on dietary acrylamide supporting a difference in the associations by sex, and this analysis needs to be replicated.

In our analysis, we only observed a mild decline of cognition with dietary exposure of acrylamide. The modest changes could be due to the relatively low acrylamide exposure from diet, the relatively normal cognitive status of our participants or the inadequate follow-up period for development of impaired cognition. Most of our participants had a relatively normal cognition, with average scores at baseline of being 25, and 22.4% (568/2534) of participants reaching the maximum score of 29. In addition, a learning effect at second test may lead to the modest change of cognition. The combination of the ceiling and learning effects may thus attenuate the inverse association that we found between acrylamide levels and cognition. Moreover, we follow the cognition change only for 4 years period. It may be important to study the longer term effects of dietary acrylamide intake on risk of poor cognition. However, currently there is no evidence to provide the possible biological latent.

We additionally showed that adjusting TL for the regression model in men weakened the association of acrylamide exposure and cognition. This suggests that TL may mediate the association. Acrylamide and its metabolite glycidamide are reactive and may form adducts with nucleophilic sites in proteins and DNA^31^. Telomeres are regions of repetitive DNA at the ends of chromosomes that protect from DNA rearrangements and chromosomal end-to-end fusions and have established roles in biological aging^32^. Shorter leukocyte TL has been reported to be associated with age-related diseases including cognitive impairment^33^. It is hypothesized that TL attrition is associated with the cognitive decline, which might reflect the consequences of increased oxidative stress^15^.

The proposed mechanisms for acrylamide neurotoxicity on cognitive function include central nerve terminal degeneration, harmful effects on the cerebral cortex, thalamus and hippocampus, direct inhibition of neurotransmission by decreasing release of neurotransmitter^14^, or interference with kinesin motor protein function and nerve signal transportation^34^. A recent *in vitro* study^35^ reported that non-cytotoxic concentrations of acrylamide alter neurotransmitter induced calcium responses in murine ESC-derived and primary neurons. In addition, acrylamide can damage the blood-cerebro spinal fluid barrier and impairs secretory and transport functions. These changes may underlie acrylamide-induced neurotoxicity^36^. Morphologic examinations also revealed that low-dose subchronic induction of acrylamide neurotoxicity was associated with nerve damage in both the central and peripheral nervous systems^37^, which could impede the development of learning skills^10^.

Our results should be interpreted with caution due to some potential limitations. First, habitual acrylamide exposure was determined by FFQ and the measurement errors in the dietary survey may have influenced the results. Furthermore, the participants were aged 65 and older and therefore may be less reliable in recalling food intake than younger subjects, although the FFQ was validated in the elderly population and participants with impaired cognition at baseline were excluded. Moreover, when matching FFQ data (319 food items) from contamination data from the TDS (150 food items), some items did not correspond. However, the recall or information error is expected to produce non-differential misclassification and is highly unlikely to bias results away from the null, but rather to underestimate the observed associations.

Second, although MMSE is the widely used instrument for measuring the course of cognitive change in older adults over time. It may not have been sensitive enough to pick up specific or subtle cognitive deficits. Future studies using more detailed and comprehensive cognitive assessment tools to diagnose impaired cognition are necessary.

Third, as this is a volunteer based cohort, it is possible that our study participants had better physical and mental health than the elderly population as a whole. In addition, participants who were not assessed at follow-up were older, had lower mean BMI, lower cognitive performance and more depressive symptoms than participants who were followed up. Thus, they might not be representative of the general population as there could be ‘potential healthy volunteer bias’. Another limitation was that we didn’t collect the data on passive smoking although the smoke could be less concentrated to an involuntary smoker than that inhaled by smokers. Future studies on acrylamide and elderly cognition are warranted to investigate the environment tobacco smoke (ETS). Finally, as an observational design, residual confounding by unmeasured variables remains a possibility although we have investigated most of important confounders.

This study has several strengths. First, to our knowledge, this is the first prospective study to investigate the association of dietary acrylamide exposure and cognitive function in older population. Second, assessment of dietary intake through a local TDS has also strong advantages. TDS has been recognised internationally as one of the most cost effective ways to estimate dietary exposures to food chemicals for various population groups and to assess their associated health risks^18^. A unique aspect of the TDS is that foods are prepared as they would be consumed, so the analytical results provide the basis for realistic estimates of the dietary intake of these contaminants. TDS can identify a majority of potentially contaminated foods or food groups and assess contamination that may be widely distributed across the entire food supply, given its nature of covering total diet and presenting food contaminants even at a very low level. In contrast to risk assessment by biomarkers, evaluation of acrylamide from diet is noninvasive and relatively accurate which is thus regarded as a valid approach to be adopted in establishing guidelines for specific food recommendations. Previous study showed well-designed FFQ were good proxies for estimating average levels of acrylamide for non-smokers^24^. Although the use of FFQs has limitations of possible misclassification, they are the only feasible way in large epidemiological studies to assess the intake of the relevant acrylamide-containing foods over a long time period^38^. In addition, the non-differential misclassification would only bias the risk estimates toward null. Although acrylamide adducts to hemoglobin are recognized as the internal dose markers of ‘exposure’ to acrylamide^39^, they represent the exposure during the preceding 3~4 months only. The costs of using biomarkers also limit the size of the population that can be used.

## Conclusion

Our findings indicated that, dietary acrylamide exposure was associated with mild cognitive decline or increased risk of poor cognition over a 4-year period in non-smoking Chinese elderly men but not in women. Further prospective studies with longer follow-up and more comprehensive measures on cognition are warranted to clarify the adverse effect of acrylamide and the underlying mechanisms.

## Electronic supplementary material

Supplemental Tables
