# Supplementary material for: Dietary acrylamide exposure was associated with mild cognition decline among non-smoking Chinese elderly men
Source: Sci Rep. 2017 Jul 25;7:6395. doi: 10.1038/s41598-017-06813-9 (PMC5527102; doi:10.1038/s41598-017-06813-9)
Supplement: Supplementary file 1 — Supplemental Tables [file 41598_2017_6813_MOESM1_ESM.doc]

**Dietary acrylamide exposure was associated with mild cognition decline among non-smoking Chinese elderly men**

Zhao-min Liu,Ph^D,1^,2*, Lap Ah Tse,PhD,^2*^ Bailing Chen,MD,^3^ Suyang Wu,PhD^2^, Dicken Chan,MSc,^4^ Timothy Kowk,MD,^5^ Jean Woo,MD,^5^ Yu-Tao Xiang,PhD,^6^ Samuel Yeung-shan Wong,MD^4^

^1^Department of Nutrition, School of Public Health, Sun Yat-sen University, Guangzhou, China; ^2^Division of Environmental and Occupational Health, Jockey Club School of Public Health and Primary Care, the Chinese University of Hong Kong, Hong Kong SAR; ^3^Department of Spine Surgery, The First Affiliated Hospital of Sun Yat-sen University, Guangzhou, PR, China;^4^Division of Family Medicine and Primary Care, Jockey Club School of Public Health and Primary Care, the Chinese University of Hong Kong, Hong Kong SAR; ^5^Department of Medicine and Therapeutics, the Chinese University of Hong Kong, Hong Kong SAR; ^6^Unit of Psychiatry, Faculty of Health Sciences, University of Macau, Macao SAR, China.

**Supplemental Table 1 Sensitivity analyses with exclusion of participants of diabetes, stroke or cardiovascular diseases on dietary acrylamide intake and changes of MMSE at 4^th^ year follow-up among Chinese elderly men and women by multivariable linear** regression

|  | **Model 2 (full adjustment)** | **P** |
| --- | --- | --- |
| **MMSE 4-year change** | β (95% CI) |  |
| Men (n=723) | -1.881 (-0.371, -0.052) | 0.044 |
| Women (n=1809) | -0.202 (-1.317, 0.913) | 0.722 |
| Participants with MMSE≥18 |  |  |
| Men (n=718) | -1.892 (-0.381, -0.052) | 0.042 |
| Women (n=1709 ) | -0.403 (-1.508, 0.702) | 0.474 |

Dietary acrylamide intakes were log_10_ transformed. Multivariable linear regression models were adjusted for age (y), education, income, physical activity (PASE total scores), body mass index (kg/m^2^), dietary intake of carbonhydrate (g%kcal), fish (g/week), fruits and vegetables (g/1000kcal), fiber (g/d) and isoflavones (mg/d), alcohol drinking (g/day), tea drinking (ml/wk), total AHA scores. Total AHA scores were estimated based on the adherence index of American Heart Association on dietary and life style recommendations. MMSE: questionnaire for Mini-Mental State Exam.

**Supplemental Table 2 Sensitivity analyses with exclusion of participants of diabetes, stroke or cardiovascular diseases on dietary acrylamide intake and risk of poor cognition (MMSE≤26) among Chinese elderly men and women by multivariable logistic regression**

|  |  | **Full adjusted model** | **P** |
| --- | --- | --- | --- |
|  |  | Hazard ratios (95% CI) |  |
| **4^th^ y MMSE≤26 as cutoff** |  |  |  |
| Men (n=468) |  | 3.677 (1.029, 13.142) | 0.045 |
| Women (n=1148) |  | 1.059 (0.473, 2.369) | 0.831 |

Data analysis was conducted by logistic regression model. Hazard ratios: Risk of MMSE≤26 with an increase of 1 µg/d acrylamide intake. Adjusted variables included age (y), education, PASE total scores, dietary carbohydrate intake (% total energy), total AHA scores, baseline body weight, coffee (ml/d), tea drinking (ml/d), alcohol drinking (ml/d), dietary total isoflavones intake (mg/d), fruits and vegetables intake (g/1000kcal), fish consumption (g/1000kcal). Total AHA scores were estimated based on the adherence index of American Heart Association on dietary and life style recommendations. MMSE: questionnaire for Mini-Mental State Exam.

**Supplemental Table 3 Worst-case analysis by exclusion of participants with acrylamide intake above 95% CI on the association of dietary acrylamide exposure and the changes of MMSE at 4^th^ year follow-up among non-smoking Chinese elderly men and women**

|  | **Full adjusted model** | **P** |
| --- | --- | --- |
| **MMSE 4-year change** | β (95% CI) |  |
| Men (n=671) | -1.228 (-2.827, 0.371) | 0.132 |
| Women (n=1734) | -0.324 (-1.331, 0.682) | 0.528 |
| Participants with MMSE≥18 |  |  |
| Men (n=666) | -1.139 (-2.731, 0.452) | 0.160 |
| Women (n=1640 ) | -0.706 (-1.707, 0.295) | 0.167 |
| **MMSE 4-year % change** |  |  |
| Men (n=548) | -4.408 (-10.723, 1.906) | 0.171 |
| Women (n=1385) | -1.264 (-5.958, 3.430) | 0.597 |
| Participants with MMSE≥18 (n=1967) |  |  |
| Men (n= 547) | -4.443 (-12.733, 3.847) | 0.293 |
| Women (n=1328 ) | -2.867 (-7.577, 1.843) | 0.233 |

Dietary acrylamide intakes were log_10_ transformed. Multi-variable linear regression models were adjusted for age (y), education, income, physical activity (PASE total scores), body mass index (kg/m^2^), medical history of hypertension (yes/no), diabetes (yes/no), and coronary heart disease (CHD)(yes/no), dietary intake of carbonhydrate (g%kcal), fish (g/week), fruit and vegetables (g/1000kcal), fiber (g/d) and isoflavones (mg/d), alcohol drinking (g/day), tea drinking (ml/wk), total AHA scores. Total AHA scores were estimated based on the adherence index of American Heart Association on dietary and life style recommendations. MMSE: questionnaire for Mini-Mental State Exam.

**Supplemental Table 4 Worst-case analysis by exclusion of participants with acrylamide intake above 95% CI on hazard ratios (95% CI) of impaired cognition (MMSE≤26) at 4^th^ year follow-up by** logistic regression among non-smoking Chinese elderly men and women

|  |  | **Full adjusted model** | **P** |
| --- | --- | --- | --- |
|  |  | HR (95% CI) |  |
| **4^th^ y MMSE≤26 as cutoff** |  |  |  |
| Men (n=671) |  | 2.032 (0.505, 8.184) | 0.318 |
| Women (n=1148) |  | 1.082 (0.646, 2.629) | 0.246 |

Data analysis was conducted by logistic regression model by exclusion of participants with acrylamide intake above 95% CI. Hazard rations (HR): Risk of MMSE≤26 with an increase of 1 µg/d acrylamide intake. Adjusted variables included age (y), education, PASE total scores, dietary carbohydrate intake (% total energy), total AHA scores, baseline body weight, coffee (ml/d), tea drinking (ml/d), alcohol drinking (ml/d), medical history of diabetes (yes/no), stroke (yes/no), hypertension (yes/no), heart infarction (yes/no), any cancers (yes/no), total isoflavoens intake (mg/d), fruit and vegetables intakes (g/1000kcal), fish consumption (g/1000kcal). Total AHA scores were estimated based on the adherence index of American Heart Association on dietary and life style recommendations. MMSE: questionnaire for Mini-Mental State Exam.
